# Revisiting Identification Thresholds of Specific Learning Disorders (SLD) in Gifted Children (GC). A Comparison of Three Groups: Twice‐Exceptional (TE) Children, GC Without SLD and Non‐GC With SLD

**DOI:** 10.1002/dys.70050

**Published:** 2026-10-15

**Authors:** Laurent Lesecq, Laurent Querne, Julie Gornes, Laura Buffo, Louise Corbel, Loïse Perrais, Maëlys Kartner, Anne Gaelle Le Moing, Beatrice Bourdin, Patrick Berquin

**Affiliations:** ^1^ Department of Pediatric Neurology and Referral Center for Language and Learning Disorders (CRTLA) Amiens‐Picardie University Medical Center Amiens France; ^2^ Psychology Research Center—Cognition, Psychism, Organisations, PRC‐CPO (EA7273) Jules Verne University of Picardie Amiens France; ^3^ Groupe de Recherches Sur L'analyse Multimodale de la Fonction Cérébrale (GRAMFC) INSERM U1105 Amiens France; ^4^ University Department of Education and Training in Speech Therapy Paul Sabatier University Toulouse France

**Keywords:** dyslexia, gifted child, identification threshold, language opacity, specific learning disorder, twice exceptional

## Abstract

This study investigates assessment of Specific Learning Disorders (SLD) in Twice‐Exceptional (TE) children, gifted children (GC) who also present SLD, in oral and written language domains. Applying the standard threshold for identification (< 7th percentile) to GC often results in the assessment and identification of SLD in the TE population, thereby limiting access to appropriate support. The objective was to determine which language tests effectively identify SLD in TE children and establish optimal threshold values. The prospective study included 301 children 7–16 years, divided into three groups: GC without SLD (*n* = 71), TE children (*n* = 114), and non‐gifted children with SLD (*n* = 116). Language test performances were compared across groups, and Classification and Regression Tree (CART) analyses were employed to optimize test sensitivity and specificity in the TE group. TE children performed better than the SLD group but worse than GC without disorders. At standard thresholds, text reading and spelling tests showed the highest sensitivity, which significantly improved when CART‐derived decision thresholds (62nd and 38th percentiles) were applied, while maintaining satisfactory specificity. Results indicate that standard thresholds lead to under‐identification of SLD in GC, and that adjusted thresholds enhance identification accuracy. These findings should generalize to similarly complex languages, such as English.

**Abbreviations**
CART: classification and regression tree analyses
FSIQ: full scale intelligence quotient
GC: gifted children
HRI: highest score for the reasoning indexes
SLD: specific learning disabilities
TE: twice‐exceptional children

## 1 Introduction

In recent years, growing interest has emerged around Gifted Children (GC), both in research and public discourse. Despite their high cognitive potential, some GC may present learning difficulties, including neurodevelopmental disorders such as Specific Learning Disorders (SLD), which may affect reading, written expression, and/or mathematical skills, either in isolation or comorbidly. Children who present both giftedness (or advanced academically) and a neurodevelopmental disorder are referred to in the literature as *Twice Exceptional* (TE) (Maddocks 2020). Among these neurodevelopmental disorders are specific learning disorders, which, when combined with giftedness, are the focus of the present study.

Brody and Mills (1997) and Maddocks (2018) identify three scenarios of missed identification: when giftedness is recognized but SLD is overlooked; when SLD is identified but giftedness is unrecognized; and when both conditions remain undetected. This dual exceptionality is frequently overlooked and under‐identified, restricting access to specialized interventions and tailored educational support. Additionally, failure to recognize SLD in these children may lead to psychological challenges that adversely affect their academic outcomes (Desvaux et al. 2023).

Giftedness, not being a pathology or disorder, is more appropriately described through identification rather than diagnosis (Brasseur and Cuche 2017). According to the American Psychological Association (n.d.), giftedness is frequently characterized as a developmental condition defined by significantly high inherent intellectual capacity that usually manifests early in life. A Full‐Scale IQ of roughly 130 or higher, which is roughly two standard deviations above the mean and represents about 2.5% of the population on the majority of standardized intelligence tests, is frequently used to operationalize intellectual giftedness in psychometric terms (American Psychological Association n.d.; Wechsler, as cited in traditional classification practices). However, this cutoff is not universally agreed upon and varies across cultural and theoretical contexts (Bourdin and Aubry 2024). Some researchers suggest lower thresholds (120–125), while others recommend higher ones (135–140) (Freeman 1980; Tordjman and Kermarrec 2019; Wahl and Wahl 2020; Vaivre‐Douret 2021).

Definitions, causes, and manifestations of SLD remain debated, particularly due to language‐dependent variations. According to Diagnostic and Statistical Manual of Mental Disorders (DSM‐5) (5th ed.; American Psychiatric Association 2015), SLD involve persistent academic difficulties, with identification based on significantly low performance (typically ≥ 1.5 SD below age norms, below the 7th percentile) on standardized tests. Cutoff scores may vary by assessment, and clinical judgement can adjust thresholds when supported by converging evidence. These difficulties must persist for at least 6 months despite targeted interventions.

The relationship between reading performance and higher cognitive abilities, including compensatory mechanisms, remains debated in the literature. Some authors (Lijbers et al. 2025) argue that GC are generally unable to compensate for dyslexia despite their superior cognitive skills, and that proposed compensatory factors (e.g., memory, vocabulary) lack strong empirical support. Phonological awareness, rapid automatized naming (RAN), and non‐word repetition are key underlying cognitive skills for literacy acquisition. While research links these abilities to the risk of dyslexia, they are neither necessary nor sufficient for diagnosis. Difficulties in these areas may increase the risk of severe reading and spelling difficulties, but their presence alone does not warrant an identification of dyslexia, nor does their absence rule it out (Pennington and Bishop 2009; Pennington and Peterson 2012; Snowling 2008). Other authors suggest that gifted children may outperform their non‐gifted peers with SLD in foundational reading‐related skills, such as those previously mentioned (Berninger and Abbott 2013; Bucaille et al. 2022; Kranz et al. 2024; van Viersen et al. 2016). These scores, often within the average range, may therefore mask underlying learning disorder markers for clinicians. Conventional assessment tools frequently fail to identify SLD in GC due to masking effects, reliance on normative comparisons, and a lack of intra‐individual performance analysis (Maddocks 2018; Kranz et al. 2024: Lesecq et al. 2024). As a result, learning disorders may remain undetected until academic demands exceed the child's compensatory capacities (Terriot 2021).

The oral and written language abilities of GC remain largely underexplored. As emphasized in the recent review by Kranz et al. (2024), the literature provides limited data on this topic. The authors underscored the urgent need for research comparing GC with and without SLD to better characterize their language and reading profiles, thereby facilitating more accurate identification of SLD. These findings raise important questions regarding the appropriateness of applying conventional cut‐off criteria to oral and written language tests in the GC population.

Consistent with findings from recent reviews (Desvaux et al. 2023; Kranz et al. 2024), it is reasonable to assume that gifted children (GC) would have a higher or less severe performance threshold for SLD than the general population. This hypothesis is supported by the finding that GC typically have better language skills than their peers of the same age (van Viersen et al. 2016). Thus, a language test score that would be regarded as “within the norm” for the general population may really indicate a relative discrepancy within the gifted group. In another way, if the criteria are not modified to account for the unique linguistic profile of gifted children, identification instruments and thresholds that are standardized for the general population run the danger of underestimating the presence of SLD in these children. To address this, we first evaluated the sensitivity of standard oral and written language tests for identifying language difficulties in a group of French‐speaking TE children using thresholds established for the general population.

The validity of these thresholds was further assessed in a comparison group of non‐gifted children with SLD. Second, we employed Classification and Regression Tree (CART) analyses to determine the optimal cut‐off scores that best discriminate between the TE group and a control group of children without SLD, maximizing the balance between sensitivity and specificity.

## 2 Method

### 2.1 Participants

Groups were formed as follows: GC without SLD (GC‐group, *n* = 71), GC with SLD (TE‐group, *n* = 114), and non‐gifted children with SLD (SLD‐group, *n* = 116). Children with psychiatric or neurological disorders, disorders of intellectual development or scoring below 70 on the full‐scale intelligence quotient (FSIQ) were not included in the study.

The GC‐group had no academic delay, ongoing or past school learning difficulties. They were recruited from specific classes for GC in private schools.

Data for the TE‐group and the SLD‐group were obtained from the via French speech‐therapists. All data transmitted was anonymized before communication.

The TE‐group included GC identified with a SLD by a speech‐therapist according to the DSM‐5 criteria: SLD with impairment in reading (315.00) and/or impairment in written expression (315.2).

SLD‐group included children who did not meet the criteria for giftedness as previously defined and who were identified with SLD according to the DSM‐5 criteria with impairment in reading (315.00) and/or impairment in written expression (315.2) by a speech‐therapist.

Identification criteria for giftedness are not subject to consensus in the literature (Dai and Gauvrit 2021). Therefore, we adopted a gradated inclusion approach: first, using a Full‐Scale IQ (FSIQ) above 130, as traditionally recommended in the literature (Maddocks 2020); second, the General Ability Index (GAI), which has been proposed as a relevant alternative when the FSIQ is not interpretable, particularly in the presence of discrepancies between cognitive indices (Tordjman and Kermarrec 2019); and finally the Highest score for the Reasoning Indexes (HRI) in line with the recommendations of a number of researchers (Goldschmidt 2017; Maddocks 2020; Grégoire 2021; Silverman and Gilman 2020), only children who scored 130 or more on at least one of the following reasoning indexes were included in the study: verbal‐comprehension index (VCI), visual–spatial index (VSI) and fluid‐reasoning index (FRI) for the Wechsler Intelligence Scale for Children, 5th edition (WISC‐V) and Wechsler Preschool and Primary Scale of Intelligence, 4th edition (WPPSI‐IV) and the VCI and the perceptual‐reasoning index (PRI) for the WISC, 4th edition (WISC‐IV). The highest score for the reasoning indexes (HRI) was calculated for each child.

### 2.2 Material

#### 2.2.1 Psychometric Batteries

All children completed the WISC‐IV (Wechsler 2005), the WPPSI‐IV (Wechsler 2014), or the WISC‐V (Wechsler 2016). While the WISC‐IV and WISC‐V are meant for children between the ages of 6 and 16 years 11 months, the WPPSI‐IV is designed to evaluate children between the ages of 2 years 6 months and 7 years 7 months (see Table SI.1.1: Distribution of psychometric batteries completed by children in the GC, TE, and SLD Groups (*N* and percentages) of Supporting Information (SI) SI.1). Subtests usually exhibit good reliability (often > 0.80), and these tests show strong internal consistency and test–retest reliability for composite scores. Additionally, they have shown contemporaneous validity with other cognitive and achievement measures and construct validity through factor‐analytic research. Stratified sampling was used to standardize the tests on a large, representative sample.

#### 2.2.2 Language‐Test Batteries

In France, standardized testing by speech‐therapists can legally be based on several batteries assessing language skills. For this study, patient data was chosen to include results from language‐tests assessing the main oral‐ and written‐language domains, in accordance with the guidelines for good practice in the assessment, prevention and remediation of written‐language disorders published by the French College of Speech Therapy (Leloup et al. 2022). These included tasks assessing metaphonology, rapid‐automatic‐naming (RAN), repetition, reading, and spelling. These language domains were assessed with the following language‐test batteries EVAluation du Langage Ecrit et du langage Oral [Assessment of written and oral language] (EVALEO) (Launay et al. 2018), Bilan Analytique du Langage Ecrit [Analytical review of written language] (BALE) (Jacquier‐Roux et al. 2010) and EXAmen de LANGage [Language assessment] (EXALANG) (Lenfant et al. 2009). The tests selected for the study assess reading and spelling, as well as the foundational skills for written language acquisition, such as phonology, working memory, and rapid automatized naming. The GC group (*n* = 71) exclusively took the EVALEO test. In the TE group (*n* = 114), 63 participants took the EVALEO test, 33 took the EXALANG test, and 16 took the BALE test. In the SLD group, 94 participants took the EVALEO test, 19 took the EXALANG test, and 3 took the BALE test (see Supporting Information SI.2 for the distribution of participants across oral and written language batteries (Table SI.2.1)).

Data have been collected only for the tests shared across the batteries used. These tests were as follows: phoneme‐deletion, RAN, word‐repetition, pseudoword‐repetition, word‐reading and pseudoword‐reading, text‐reading‐accuracy and spelling‐accuracy. Children in the GC group performed all these tests. Among the TE‐ and SLD‐groups, not all children had completed all the tests (Table 1), and the tests administered could have come from one or more language‐test batteries.

**TABLE 1:** Sample size, age and class‐level range of language‐test batteries.

	All sample size	Mean sample size by grade	Age range	Grade range
EVALEO	1324	145	6–15 years	First grade primary school—Ninth grade secondary school
EXALANG	747	93	5–15 years	Kindergarten—Ninth grade secondary school
BALE	665	110	7–13 years	Second grade primary school—Seven grade secondary school

*Note:* Phoneme‐deletion, rapid‐automatic‐naming, word‐repetition, pseudoword‐repetition, speed and accuracy word‐reading, speed and accuracy pseudoword‐reading, text‐reading and spelling‐accuracy are the tests common to the three language‐test batteries.

## 3 Statistical Analysis

### 3.1 Group Comparisons for Age, Sex‐Ratio and Psychometry

Age and sex‐ratio were compared between groups using the Mann–Whitney and Fisher‐exact tests, respectively. Psychometric profiles between VCI, VSI/PRI, working‐memory index (WMI) and processing‐speed index (PSI) were tested independently for each group by applying the Friedman‐test for paired data. Any differences between (i) GC‐ and TE‐groups, and (ii) TE‐ and SLD‐groups were tested for significance using the Mann–Whitney for VCI, VSI/PRI, WMI, PSI, FSIQ and HRI. Data are reported as median; inter‐quartile range [IQR] (Table 2).

**TABLE 2:** Demographic and psychometric characteristics of the GC‐group, TE‐group and SLD‐group.

	GC		TE		SLD		Pairwise comparisons		
	M	F	M	F	M	F	GC vs. TE	TE vs. SLD	GC vs. SLD
*N*	50	22	90	24	85	31			
Sex ratio	69.4%		78.9%		73.3%		ns	ns	ns

	GC		TE		SLD				
	Median	IQR	Median	IQR	Median	IQR			
Age	9.9	4.0	9.9	2.7	9.8	2.3	ns	ns	ns
HRI	139.5	13.0	141.0	10.5	103.0	14.0	ns	< 0.0001	< 0.0001
FSIQ	136.5	10.0	126.0	17.0	91.5	11.5	< 0.0001	< 0.0001	< 0.0001
VCI	137.0	14.0	139.0	13.0	97.0	17.0	ns	< 0.0001	< 0.0001
VSI/PRI	128.0	21.0	120.5	23.0	97.0	16.0	0.0012	< 0.0001	< 0.0001
WMI	128.5	16.5	106.5	24.0	88.0	15.0	< 0.0001	< 0.0001	< 0.0001
PSI	118.0	15.0	102.0	21.5	95.5	17.0	< 0.0001	0.0005	< 0.0001

*Note: p*‐Values reported after the Benjamini–Hochberg correction for multiple comparisons.

Sex ratio = male/(male + female).

Abbreviations: F, female; FSIQ, full‐scale intelligence quotient; GC, gifted children; HRI, highest score for reasoning indexes; IQR, interquartile range; M, male; PSI, processing‐speed index; SLD, specific learning disorder; TE, twice‐exceptional; VCI, visual‐spatial index; VSI/PRI, visual‐spatial index or perceptual‐reasoning index; WMI, working‐memory index.

### 3.2 Harmonization for Language‐Test Scores

Since norms were not expressed in means and SDs for all language‐batteries, scores were harmonized relative to the lowest common denominator: the EVALEO. Thus, all scores were transformed into EVALEO percentile intervals, ranked from S1 (score < 7th‐percentile), S2 (7th–20th‐percentile), S3 (21st–38th‐percentile), S4 (39th–62nd‐percentile), S5 (63rd–80th‐percentile), S6 (81st–93rd‐percentile) to S7 (>93rd‐percentile). Details of the conversion of EXALANG and BALE scores into the seven EVALEO percentile rank scores are presented in SI.2. (see Table SI.2.2: Conversion of EXALANG and BALE *Z*‐scores into the seven EVALEO percentile rank scores).

### 3.3 Group Comparisons for Language‐Tests

The performance profiles on the language‐tests for children in the three groups were compared to the standardized‐norms for each battery. The number of children who scored S1, those who scored S2, S3, S4, and S5 (aggregated into S2345), and those who scored S6 and S7 (aggregated into S67) were counted and compared to the standardized distribution of results for language‐test batteries by applying Fisher‐exact tests. The normative properties of the language‐test batteries show that among the children who participated in their standardization, 7% scored S1 (< 7th percentile), 73% scored S2345 (7th to 80th percentile), and 20% scored S67 (> 80th percentile). To account for the normative properties of test batteries, the counts for the standard‐norms of S1, S2345, and S67 were calculated proportionally, based on the average number of children per age group for each test instrument (see Table 1 for explanation).

S1‐S2345‐S67 distributions for language‐tests in the TE‐group were compared with (i) the GC‐group and (ii) the SLD‐group using the Fisher‐exact test.

### 3.4 CART

To determine the specific thresholds achieving the best sensitivity/specificity balance for distinguishing between children of the TE‐group and those of the GC‐group, we applied CART algorithms independently for each language‐test (Breiman et al. 2017). CART is a recursive partitioning procedure that determines optimal cut‐off points for a variable or set of variables (scores from S1 to S7 language‐test in this study), to maximally separate the two populations of interest: children of the TE‐group and children of the GC‐group. CART algorithm was detail in Supporting Information SI.3.

### 3.5 Correction for Multiple Comparisons

The alpha risk inflation for multiple comparisons was controlled by applying the Benjamini–Hochberg correction (all statistical‐tests were considered: *k* = 76, *p* < 0.05). All *p*‐values reported in the manuscript correspond to values obtained after the Benjamini–Hochberg correction.

Statistics were performed with R v4.3.3 (R Foundation for Statistical Computing, Vienna, Austria; https://www.r‐project.org), CART with R packages “Partykit” (Hothorn and Zeileis 2015) and “rpart” (Therneau and Atkinson 2024).

## 4 Results

### 4.1 Sample Size, Sex‐Ratio and Age

The GC‐group consisted of 71 children (49 boys, 22 girls [sex‐ratio = 69.0%]), aged 7–15 years (median, [IQR] = 9.9, [4.0]); the TE‐group was composed of 114 children (90 boys, 24 girls [sex‐ratio = 78.9%]), aged 7–15 years (9.9, [2.7]); and the SLD‐group included 116 children (85 boys, 31 girls [sex‐ratio = 73.3%]), aged 7–16 years (9.8, [2.3]) (Table 2). The differences between groups for age and sex‐ratio were not statistically significant.

### 4.2 Psychometric Index

The GC‐group showed a decreasing cascade from the VCI (median, [IQR] = 137.0, [14.0]) to the VSI/PRI (128.5, [21.0]) and the WMI (128.5, [16.0]) and finally to the PSI (118.5, [15.0]) (W‐Kendal = 0.295, *p* < 0.0001) (Table 2, Figure 1). The TE‐group showed a more pronounced cascade from VCI (139.0, [13.0]) to VSI/PRI (120.5, [23.0]), then to WMI (106.5, [24.0]) and finally to PSI (102.0, [21.5]) (W‐Kendal = 0.569, *p* < 0.0001). In contrast, the SLD‐group exhibits a relatively flat psychometric profile (VCI = 97.0, [17.0]; VSI/PRI = 97.0, [16.0]; WMI = 88.0, [15.0]; PSI = 95.5, [17.0]; W‐Kendal = 0.16, *p* < 0.0001).

**FIGURE 1:**
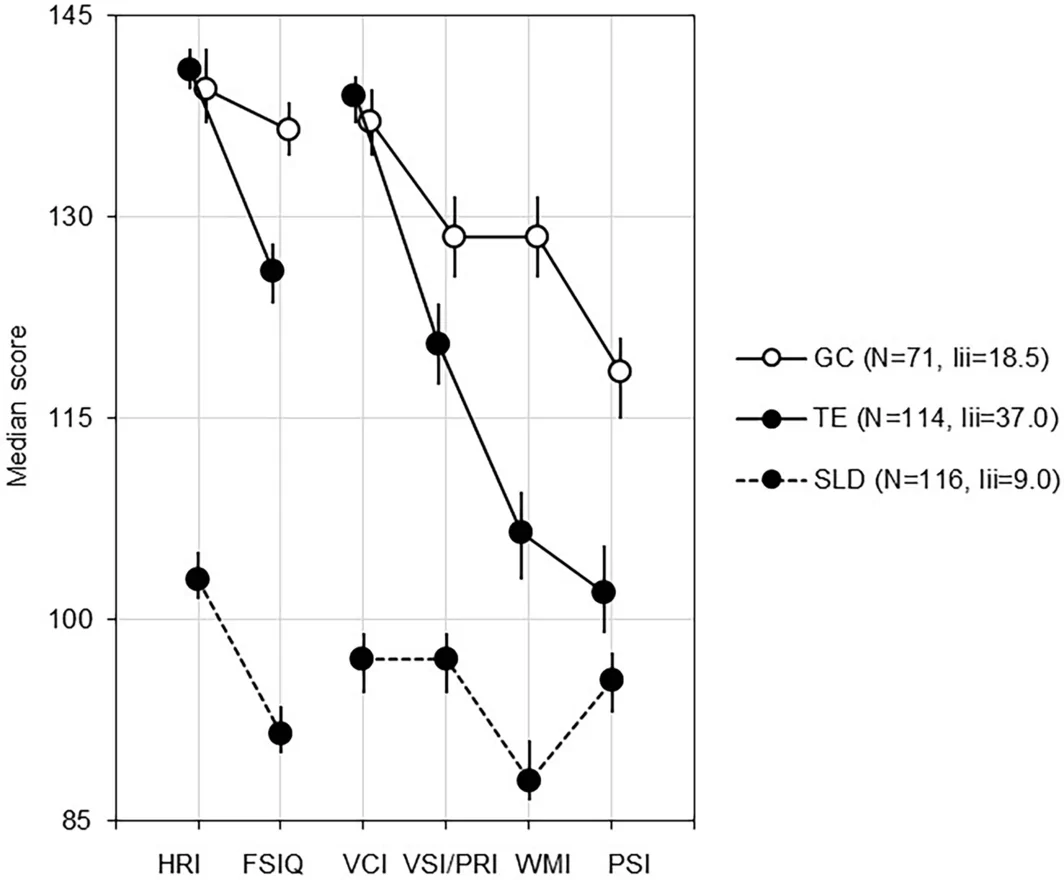
Psychometric profiles with median and 95% confidence‐interval in children from the GC‐group, the TE‐group and the SLD‐group. FSIQ, full‐scale intelligence quotient; GC, gifted children; HRI, highest‐reasoning index; Iii, inter‐index interval; *N*, sample size; PSI, processing‐speed index; SLD, specific learning disorder; TE, twice‐exceptional; VCI, verbal‐comprehension index; VSI/PRI, visual–spatial index/perceptual‐reasoning index; WMI, working‐memory index.

Group comparisons for HRI between the GC‐group (median, [IQR] = 139.5, [13.0]) and the TE‐group (141.0, [10.5]) were not statistically significant; nor were differences in VCI results. Groups differed significantly (*p* ≤ 0.0009) for VSI/PRI, WMI, PSI and FSIQ (GC‐group = 136.5, [10.5]; TE‐group = 126.0, [17.0]). The TE‐ and SLD‐groups were significantly different (*p* ≤ 0.0005) in terms of HRI (TE‐group = 141.0, [10.5]; SLD‐group = 103.0, [14.0]), FSIQ (TE‐group = 126.0, [17.0]; SLD‐group = 91.5, [11.5]) and VC1, VSI/PRI, WMI and PSI. The ratio of children with a FSIQ < 130 was 16.9% in the GC‐group and 63.2% in the TE‐group.

### 4.3 Score Distributions on Language‐Tests

*GC‐group*: Scores were significantly better than standardized norms on all language tests (*p* ≤ 0.046) except for pseudoword‐speed reading and word reading accuracy (Table 3, Figure 2).

**TABLE 3:** Score distribution in GC‐group, TE‐group and SLD‐group for language tests, and comparisons between groups and standardized norms.

	Norms	GC				Pairwise comparisons	
	N1′	N1	S1	S2345	S67	GC vs. Norms	
Phoneme‐deletion	145	72	2.8%	62.5%	34.7%	ns	
Rapid‐automatic‐naming	145	71	4.2%	56.3%	39.4%	0.012	
Word‐repetition	145	72	0.0%	23.6%	76.4%	< 0.0001	
Pseudoword‐repetition	145	72	0.0%	58.3%	41.7%	0.0006	
Word‐reading‐speed	145	72	0.0%	65.3%	34.7%	0.0070	
Pseudoword‐reading‐speed	145	71	8.5%	78.9%	12.7%	ns	
Word‐reading‐accuracy	145	72	1.4%	66.7%	31.9%	ns	
Pseudoword‐reading‐accuracy	145	71	1.4%	64.8%	33.8%	0.037	
Text‐reading‐accuracy	145	72	0.0%	68.1%	31.9%	0.0038	
Spelling‐accuracy	145	71	1.4%	58.3%	40.3%	0.012	

	Norms	GC				Pairwise comparisons	
	N2′	N2	S1	S2345	S67	TE vs. Norms	TE vs. GC
Phoneme‐deletion	93	64	36.4%	52.7%	10.9%	< 0.0001	< 0.0001
Rapid‐automatic‐naming	93	55	42.2%	54.7%	3.1%	< 0.0001	< 0.0001
Word‐repetition	93	59	15.3%	66.1%	18.6%	ns	< 0.0001
Pseudoword‐repetition	93	65	21.5%	66.2%	12.3%	0.020	< 0.0001
Word‐reading‐speed	93	99	45.5%	49.5%	5.1%	< 0.0001	< 0.0001
Pseudoword‐reading‐speed	93	96	50.0%	46.9%	3.1%	< 0.0001	< 0.0001
Word‐reading‐accuracy	93	100	54.0%	43.0%	3.0%	< 0.0001	< 0.0001
Pseudoword‐reading‐accuracy	93	97	57.7%	42.3%	0.0%	< 0.0001	< 0.0001
Text‐reading‐accuracy	93	87	60.9%	37.9%	1.1%	< 0.0001	< 0.0001
Spelling‐accuracy	93	108	77.8%	22.2%	0.0%	< 0.0001	< 0.0001

	Norms	GC				Pairwise comparisons	
	N3	N3′	S1	S2345	S67	SLD vs. Norms	SLD vs. TE
Phoneme‐deletion	93	101	80.2%	19.8%	0.0%	< 0.0001	< 0.0001
Rapid‐automatic‐naming	93	81	46.5%	46.5%	6.9%	< 0.0001	ns
Word‐repetition	93	107	61.7%	38.3%	0.0%	< 0.0001	< 0.0001
Pseudoword‐repetition	93	108	71.3%	26.9%	1.9%	< 0.0001	< 0.0001
Word‐reading‐speed	93	116	81.0%	19.0%	0.0%	< 0.0001	< 0.0001
Pseudoword‐reading‐speed	93	109	88.1%	11.9%	0.0%	< 0.0001	< 0.0001
Word‐reading‐accuracy	93	116	85.3%	14.7%	0.0%	< 0.0001	< 0.0001
Pseudoword‐reading‐accuracy	93	109	89.0%	11.0%	0.0%	< 0.0001	< 0.0001
Text‐reading‐accuracy	93	109	92.7%	7.3%	0.0%	< 0.0001	< 0.0001
Spelling‐accuracy	93	116	99.1%	0.9%	0.0%	< 0.0001	< 0.0001

*Note:* For example, the statistical test (Fisher‐exact test) conducted to compare scores obtained by the GC‐group to the standardized‐norms for phoneme‐deletion was performed by comparing the S1‐S2345‐S67 distribution of the GC (2 children scored S1, 45 scored S2345, and 25 scored S56, corresponding to 2.8%, 62.5% and 34.7% of the 45 GC [N1], respectively) with the S1‐S2345‐S67 distribution of norms (7.0% of the children of the EVALEO battery scored S1, 73.0% scored S2345, and 20.0% scored S67, corresponding to 10, 106, and 29 children among the 145 children in mean by school level for these battery [N1′], respectively). *p*‐values reported after the Benjamini–Hochberg correction for multiple comparisons.

Abbreviations: GC: gifted children; N1: GC‐group sample size; N1′: standardized‐norms sample size for GC/norms comparisons; N2: TE‐group sample size; N2′: standardized‐norms sample size for TE/norms comparisons; N3: SLD‐group sample size; N3′: standardized‐norms sample size for SLD/norms comparisons; ns: nonsignificant; SLD: specific learning disorder; TE: twice‐exceptional.

**FIGURE 2:**
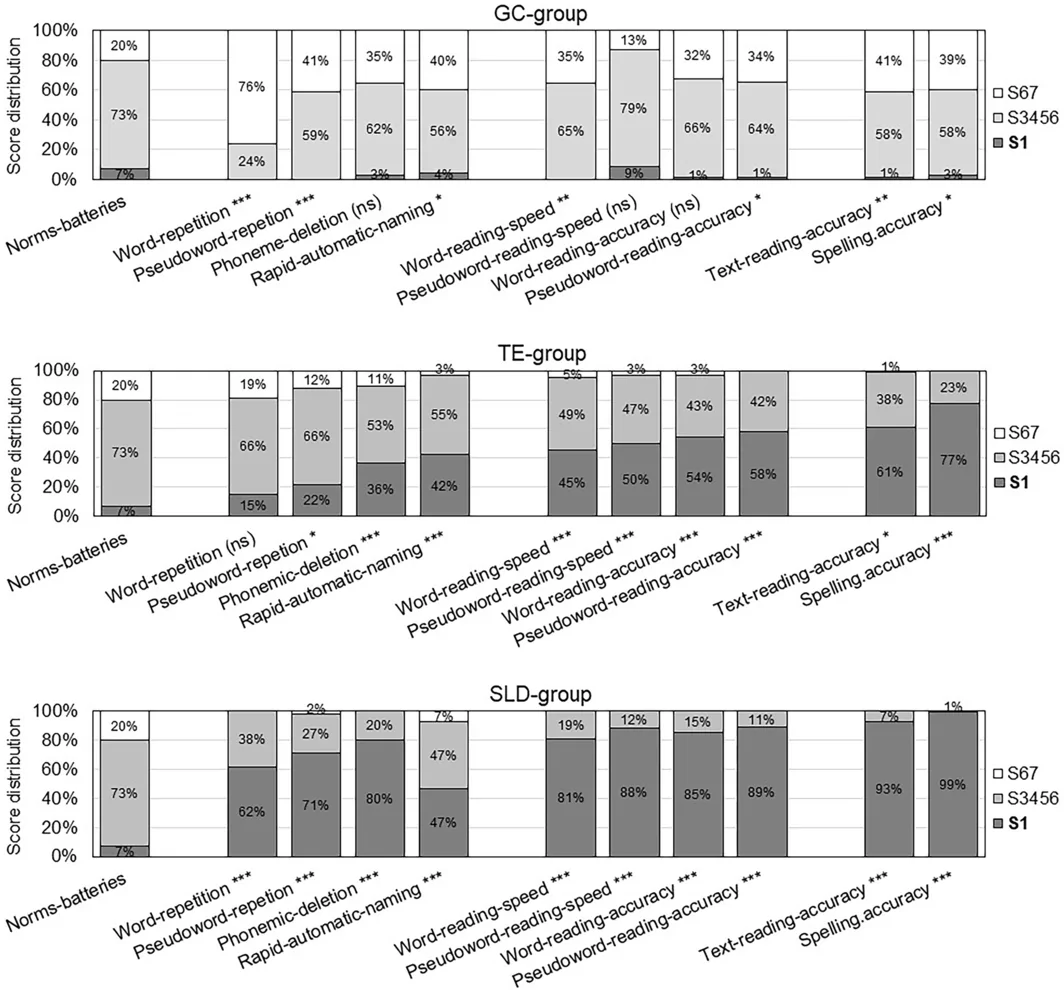
Score distributions for language‐tests in children from the GC‐group, the TE‐group and the SLD‐group. The *p*‐values indicated the language tests for which the children in the GC‐group, TE‐group, and SLD‐group obtained a score distribution that significantly differs from the standardized‐norms (***p < 0.001; **p < 0.01; *p < 0.05). GC, gifted‐children; ns, non‐significant; SLD, specific learning disorder; TE, twice‐exceptional.

*TE‐group*: Scores were significantly lower than standardized‐norms on all language‐tests (*p* ≤ 0.017), except for word‐repetition; compared to scores for the GC‐group, they were also significantly lower on all language‐tests (*p* < 0.0001).

*SLD‐group*: Scores were significantly lower than standardized norms on all language tests (*p* < 0.0001); compared to scores for the TE‐group, they were also significantly lower on all language tests (*p* < 0.0001) except on the RAN test.

#### 4.3.1 Sensitivity of Language‐Tests for SLD‐ and TE‐Groups When Applying the Standard DSM‐5 Threshold

In the SLD‐group, the ratio of children who obtained what is considered a conventional threshold score for the general population (score ≤ S1) was above 80% for all language‐tests except for RAN (46.5%), word‐repetition (61.7%) and pseudoword‐repetition (71.3%). The highest sensitivities were found for text‐reading‐accuracy (92.7%) and spelling‐accuracy (99.0%) (Table 4).

**TABLE 4:** Sensitivity of language‐tests in SLD‐ and TE‐groups when applying standard identification‐thresholds, in the TE‐group with CART‐thresholds and specificity in the GC‐group with CART‐thresholds.

	Usual threshold			CART threshold		
		Sensitivity in SLD	Sensitivity in TE		Sensitivity in TE	Specificity in GC
Phoneme‐deletion	S1	80.2%	36.4%	S2	50.9%	79.2%
Rapid‐automatic‐naming	S1	46.5%	42.2%	S4	92.2%	56.3%
Word‐repetition	S1	61.7%	15.3%	S4	91.5%	87.5%
Pseudoword‐repetition	S1	71.3%	21.5%	S4	86.2%	58.3%
Word‐reading‐speed	S1	81.0%	45.5%	S2	89.9%	91.7%
Pseudoword‐reading‐speed	S1	88.0%	50.0%	S1	50.0%	91.5%
Word‐reading‐accuracy	S1	85.3%	54.0%	S2	91.0%	94.4%
Pseudoword‐reading‐accuracy	S1	89.0%	57.7%	S3	81.4%	84.5%
Text‐reading‐accuracy	S1	92.7%	60.9%	S4	97.7%	68.1%
Spelling‐accuracy	S1	99.0%	77.8%	S3	96.3%	87.3%

*Note:* Sensibility = TP/(TP + FN); Specificity = TN/(TN + FP).

Abbreviations: CART, classification and regression tree analyses; FN, false‐negative (score > threshold in SLD or TE groups); FP, false‐positive (score ≤ threshold in the GC group); GC, gifted children; S1, score < 7th percentile; S2, score < 20th percentile; S3, score < 38th percentile; S4, score < 62nd percentile; S6, score < 93rd percentile; SLD, specific learning disorder; TE, twice‐exceptional; TN, true‐negative (score > threshold in the GC group); TP, true‐positive (score ≤ threshold in SLD or TE groups).

In the TE‐group, although sensitivities were lower than for the SLD‐group, the highest sensitivities at the standard‐threshold (score ≤ S1) were also associated with text‐reading‐accuracy (60.9%) and spelling‐accuracy (77.8%). For other language‐tests, sensitivities did not exceed 57.7%.

#### 4.3.2 CART‐Defined Thresholds to Optimize the Sensitivity/Specificity Balance Between TE‐ and GC‐Groups

The threshold determined by CART algorithms that best split children between the TE‐group and the GC‐group were higher than S1 for all tests except for the pseudoword‐speed‐reading test (Table 4). Applying to the CART‐thresholds, the ratio of TE children who obtained a pathological score was greater than 80% for all tests, except for the phoneme‐deletion (50.9%), word‐reading‐accuracy (64.0%) and pseudoword‐speed‐reading (50.0%). The highest sensitivities with CART‐thresholds were obtained for text‐reading‐accuracy (97.7%) and spelling‐accuracy (96.3%), with thresholds lower than or equal to S4 and S3, respectively. At these thresholds, the specificity obtained relative to the GC‐group was 69.0% for text‐reading‐accuracy and 88.6% for spelling‐accuracy (Figure 3).

**FIGURE 3:**
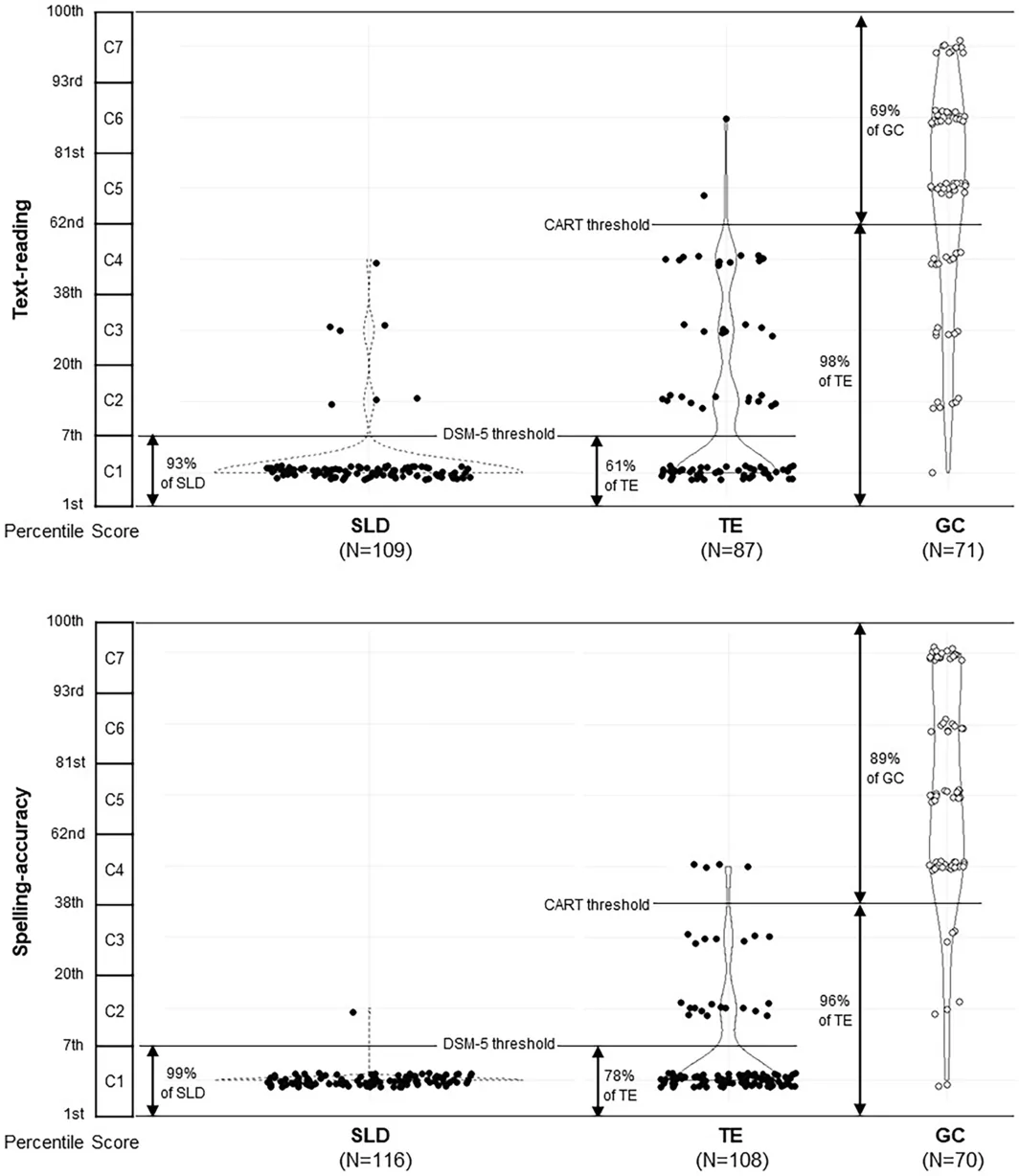
Distribution of children in the SLD‐group, the TE‐group and the GC‐group on either side of the standard DSM‐5 identification threshold or the CART‐defined threshold for text‐reading and spelling‐accuracy. CART, classification and regression tree analyses; GC, gifted‐children; SLD, specific learning disorder; TE, twice‐exceptional. *N*: sample‐size of children who realized the language‐test considered in a group. A jitter function was applied to the plots to allow children achieving same scores on a given language‐test to be distinguished on the graph.

Additional information concerning impairment across language domains in the TE and SLD groups relative to the general population (using a Relative Risk [RR] approach) was examined. A statistically significant linear regression (*p* < 0.001) of RRs between the TE and SLD groups indicated a graded pattern of impairment across language domains in both groups compared to the general population: mild‐to‐moderate impairment on tests assessing prerequisite reading skills, moderately severe on reading tests, and marked‐to‐severe impairment on spelling tests. The gradient of deficit across language domains was more pronounced in the TE group than in the SLD group (linear regression slope = +1.51$), indicating that the deficit gap between the two groups diminished as one moved along the gradient (RR ratio: RR_SLD_/RR_TE_ = 2.63 for prerequisite reading skills, 1.54 for reading tests, and 1.28 for spelling tests) (see Supporting Information SI.4).

## 5 Discussion

The GC in this study performed significantly above the standard‐norms on all language‐tests except in pseudoword speed‐reading and word reading‐accuracy, while the non‐gifted children with a SLD performed significantly below the standard norms on all language‐tests. The performance of TE children (GC with a SLD for reading and/or written expression) was intermediate between the other two groups: (i) significantly lower than the GC for all language‐tests, but (ii) significantly higher than non‐GC with SLD, except in the rapid‐automatic‐naming test. In addition, TE children performed significantly below standard‐norms in all language‐tests, except for word‐repetition. These results are consistent with the single published study on the subject involving a small Dutch speaking sample (van Viersen et al. 2016) and with other well‐established data relating to non‐GC with SLD (Lefevre et al. 2023). In this population, at the usual identification threshold (scores below the 7th‐percentile), the text‐reading (93%) and spelling (99%) tests were the most sensitive. Remarkably, these same tests showed the highest sensitivity for the TE children studied here, with a sensitivity of 61% for text‐reading and 78% for spelling. These sensitivities are noticeably lower than those observed in the SLD group, indicating that applying standard identification thresholds may lead to an under‐identification of SLD in TE children. However, written language assessment should not be limited to these two domains; indeed, speech and language therapists are expected to carry out a comprehensive screening of the child's overall linguistic strengths and weaknesses.

Based on these results, over a fifth of the TE children in the sample would not meet the usual criteria for identification of SLD (7th percentile threshold). This finding supports the hypothesis that it specific thresholds must be found to correctly identified the TE population, as proposed in several recent publications (van Viersen et al. 2016; Desvaux et al. 2023; Kranz et al. 2024). This study showed that the CART algorithm is efficient to establish a identification threshold that provides a better sensitivity/specificity balance in TE children when compared to a control GC‐group. Except for phoneme‐deletion and pseudoword‐speed‐reading, where sensitivity remained poor in the TE‐group, the CART algorithm identified thresholds that yielded sensitivities exceeding 80% for all language‐tests. The highest sensitivities were once again obtained for text‐reading and spelling‐accuracy (98% and 96% for thresholds below the 62nd and the 38th percentile, respectively); a modest specificity was found for text‐reading (69%); and a satisfactory specificity was obtained for spelling (89%).

In terms of psychometric profile, the GC‐ and TE‐groups in this study exhibited the classical decreasing cascade from the VCI to the Visual Spatial/Perceptual Reasoning Index, then to the Working Memory Index and finally to the Processing Speed Index described in the literature (Liratni and Pry 2007; Labouret and Grégoire 2018). Thus, our sample appears to be quite representative of the GC population. The results obtained confirmed that there is extensive heterogeneity across psychometric profiles among GC, and even more so among TE children (differential of 18.5 points among the GC‐group and 37 among the TE‐group, compared to only 9 among the SLD‐group). The consequence of this heterogeneity is that 17% of the children in the GC‐group and 63% in the TE‐group had an FSIQ of less than 130. This finding argues against the strict use of only the FSIQ as a criterion to identify GC, particularly in children with SLD for whom psychometric heterogeneity may be high. FSIQ scores below 130 in GC and TE children could result from (i) the way this composite index includes subtests that correlate poorly with general intelligence, such as the digit span or coding subtests, and (ii) the fact that the excellent reasoning of these children may not concern all domains (Brasseur and Goldschmidt 2020) (e.g., very high scores on verbal reasoning subtests and average or moderately high scores on non‐verbal reasoning subtests in the same child will reduce their FSIQ). This extensive heterogeneity of cognitive indexes among GC and even more so in TE mirrors the data in the literature. In line with several other authors (Brasseur and Goldschmidt 2020; Toffalini et al. 2017), we therefore argue that obtaining a score of 130 or more on one of the reasoning indexes should be taken as sufficient to identify giftedness. The systematic review conducted by Bucaille et al. (2022) further emphasizes that gifted children display marked heterogeneity in their cognitive abilities across different domains. Language skills frequently emerge as their primary area of excellence, whereas non‐verbal abilities, such as visuospatial processing and working memory, tend to be comparatively less developed and generally align with levels observed in the general population. This pattern, however, contrasts with the findings of the meta‐analysis by Rodríguez‐Naveiras et al. (2019) and the study by Aubry et al. (2021), both of which concluded that gifted children outperform typically developing peers in working memory across both verbal and visuospatial modalities.

## 6 Limitations and Future Directions

Although the sample size of the study is the largest in the literature for this population, it remains relatively modest. Given that the GC‐group's cognitive profile for the WISC corresponded to that reported in other studies, the sample appears to be quite representative of the GC population.

The children in the TE and SLD groups differ in age and educational level, and they do not all complete the same assessments or standardized language test batteries, due to legal practice in France granting speech therapists the freedom to choose language assessment tests. Despite this heterogeneity of tests, we found a very good sensitivity relative to the standard thresholds for the SLD‐group, suggesting good normative values of the different test batteries. We did not assess reading comprehension because this complex cognitive activity, which encompasses several domains (decoding, vocabulary, reasoning, inference, etc.), is extremely challenging to measure with a single standardized test (Lefevre et al. 2023; Bianco 2019; Seigneuric et al. 2001; Blanc 2010).

We can also mention here the limitation regarding the recruitment of GC. It is not easy to recruit such children in France because GC, with or without disorders, do not systematically undergo psychometric assessments, the cost of which is not covered by health insurance. Gifted children who have access to IQ tests are more often from socially advantaged families. Thus, there may be a socio‐cultural bias regarding the performance observed in our study in areas such as reading, spelling, and vocabulary.

The results presented may therefore be difficult to directly generalize from the French‐language to other languages. We nevertheless suggest that our findings could be partially applicable to languages that share some linguistic similarities with French. Among Indo‐European languages, French and, even more so, English exhibit high orthographic opacity and depth (Lefevre et al. 2023; Patel et al. 2004; Landerl et al. 2013). In comparison, Dutch and German have a moderate complexity, whereas Spanish and Italian have a low complexity (Marinus et al. 2015; Duncan et al. 2013; Joshi et al. 2022). Given the high degree of irregularity in phoneme and morpheme correspondences in English, it is probable that text‐reading tests, and particularly spelling tests, will have the highest diagnostic power for SLD in English‐speaking TE children. This hypothesis should be tested in Anglophone TE children to determine the specific identification‐threshold in them.

Our study focused on the written language performance of three distinct groups and on identifying thresholds. However, SLD cannot be reduced to technical difficulty in reading. They may constitute a genuine functional disability, particularly when they contrast with otherwise strong linguistic abilities, especially in terms of vocabulary (Elbro 2010) as is the case for children in the TE group. It would therefore be appropriate to pursue this research by assessing the functional impact of SLD using standardized questionnaires, both in GC and in gifted adults with SLD.

## 7 Conclusion

The present study demonstrates that using the standard‐threshold with French language‐tests for the general population (score below the 7th‐percentile according to the DSM‐5) may greatly underestimate SLD for reading and/or written expression in TE children. The results further showed that the CART algorithm allow to determine thresholds that enhance sensitivity in the TE‐group while preserving a satisfactory level of specificity relative to the GC‐group. The best specificity/sensitivity balance was found for text‐reading and spelling tests, which also showed the best identification value for non‐GC with SLD (Seigneuric et al. 2001).

The findings of this study clearly illustrate that it does not seem appropriate to strictly apply the conventional cut‐off score established for typically developing children to GC. Our results, which position the conventional cut‐off score for TE children close to the norm for typically developing children, may be overestimated due to a recruitment bias. It will be necessary to replicate our study to confirm or refute these results. It thus seems clinically reasonable to propose a lower cut‐off score below the 20th percentile, for reading or spelling in TE children.

The results presented highlight a public health issue in France and more generally, in other French‐speaking countries, as the use of conventional cut‐off score would fail to identify SLD in a significant proportion of GC. This would lead to missed opportunities for them in terms of classroom adaptations and appropriate care. The failure to identify their learning difficulties could lead these children to suffer (i) reduced chances of academic achievement, and (ii) psychological problems that can subsequently impede their learning (Desvaux et al. 2023). It may also be necessary to adjust the identification threshold for children who do not meet the GC criteria, but who have an intellectual efficiency level above the norm for their age group (i.e., from +1 to +2 SD). This suggestion is consistent with the DSM‐5 guidelines, which recommend that the identification threshold be adjusted depending on the clinical context.

## Funding

The authors have nothing to report.

## Ethics Statement

This prospective study was approved by the institutional review board (CPP Nord‐Ouest II [Amiens, France]; reference: PI2021_843_0098). The study database was registered with the French National Data Protection Commission (Commission nationale de l'informatique et des libertés [Paris, France]; reference: 2208336_v_0).

## Conflicts of Interest

The authors declare no conflicts of interest.

## Supporting information


**Data SI:** dys70050‐sup‐0001‐Supinfo.docx. **SI.1**. Wechsler batteries complementary information.
**Table SI:1.1** Distribution of psychometric batteries completed by children in the GC, TE, and SLD Groups (N and percentages).
**Table SI:1.2** Description of subtests for the WISC‐V, WISC‐IV, WPPSI‐III and WPPSI‐IV specifying optional subtests and those required for FSIQ calculation.
**SI.2**. Oral and written language batteries complementary information.
**Table SI:2.2** Conversion of EXALANG and BALE *Z*‐scores into the seven EVALEO percentile rank scores.
**SI.3**. Methodology of Classification and Regression Trees Analysis (CART).
**SI.4**. Relative Risks for language tests in SLD and TE groups for the usual identification threshold (≤ 7th percentile).

## Data Availability

The data that support the findings of this study are available on request from the corresponding author. The data are not publicly available due to privacy or ethical restrictions.
