# Supplementary material for: Revisiting Identification Thresholds of Specific Learning Disorders (SLD) in Gifted Children (GC). A Comparison of Three Groups: Twice‐Exceptional (TE) Children, GC Without SLD and Non‐GC With SLD
Source: Dyslexia. 2026 Oct 15;32(4):e70050. doi: 10.1002/dys.70050 (PMC13670182; doi:10.1002/dys.70050)
Supplement: Supplementary file 1 [file DYS-32-e70050-s001.docx]

**Supplementary Information (SI)**

**SI.1. Wechsler batteries complementary information**

**SI.2. Oral and written language batteries complementary information**

**SI.3. Methodology of Classification and Regression Trees Analysis (CART)**

**SI.4. Relative Risks for language tests in SLD and TE groups for the usual identification threshold (≤ 7^th^ percentile)**

**SI.1. Wechsler batteries complementary information**

The table SI.1.1 details the psychometric batteries used to assess intellectual functioning among the children in the Gifted children group, the Twice-Exceptional group and the Specific Learning Disorder group without high intellectual potential.

Table SI.1.1: Distribution of psychometric batteries completed by children in the GC, TE, and SLD Groups (N and percentages)

| **Group (N)** | **Complete psychometric assessment^(1)^** | **FSIQ available for calculation** | **WISC-V** | **WISC-IV** | **WPPSI-III** | **WPPSI-IV** |
| --- | --- | --- | --- | --- | --- | --- |
| GC (71) | 6 (8.5%) | 65 (91.5%) | 62 (87.3%) | 4 (5.6%) | 4 (5.6%) | 1 (1.4%) |
| TE (114) | 1 (0.9%) | 113 (99.1%) | 53 (46.5%) | 58 (50.9%) | 1 (0.9%) | 2 (1.8%) |
| SLD (116) | 4 (3.4%) | 112 (96.6%) | 114 (98.3%) | 2 (1.7%) | 0 (0.0%) | 0 (0.0%) |

Note: ^(1)^ Complete psychometric assessment indicates the number of children who completed all subtests, including optional subtests, within the battery.

Abbreviations: GC: Gifted children; TE: Twice-Exceptional; SLD: Specific Learning Disorder; FSIQ: Full Scale Intelligence Quotient; WPPSI: Wechsler Preschool and Primary Scale of Intelligence; WISC: Wechsler Intelligence Scale for Children.

Table SI.1.2: Description of subtests for the WISC-V, WISC-IV, WPPSI-III and WPPSI-IV specifying optional subtests and those required for FSIQ calculation

|  | **WISC-V** | | **WISC-IV** | | **WPPSI-IV** | | **WPPSI-III** | |
| --- | --- | --- | --- | --- | --- | --- | --- | --- |
|  | Index | FSIQ | Index | FSIQ | Index | FSIQ | Index | FSIQ |
| **Verbal Comprehension Index (VCI)** |  |  |  |  |  |  |  |  |
| Similarities | Required | **✓** | Required | **✓** | Required | **✓** |  |  |
| Vocabulary | Required | **✓** | Required | **✓** | Optional |  | Required | **✓** |
| Information | Optional |  | Optional |  | Required | **✓** | Required |  |
| Comprehension | Optional |  | Required | **✓** | Optional |  | Optional |  |
| **Verbal Intellectual Quotient (VIQ)** |  |  |  |  |  |  |  |  |
| Word reasoning |  |  | Optional |  |  |  | Required | **✓** |
| Picture naming |  |  |  |  | Optional |  | Optional |  |
| Receptive Vocabulary |  |  |  |  | Optional |  | Required |  |
| **Visual Spatial Index (VSI)** |  |  |  |  |  |  |  |  |
| Block design | Required | **✓** | Required | **✓** | Required | **✓** | Required | **✓** |
| Visual puzzles | Required |  |  |  |  |  |  |  |
| **Fluid Reasoning Index (FRI)** |  |  |  |  |  |  |  |  |
| Matrix Reasoning | Required | **✓** | Required | **✓** | Required | **✓** | Required | **✓** |
| Figure Weights | Required | **✓** |  |  |  |  |  |  |
| Picture Concepts |  |  | Required | **✓** | Required | **✓** | Optional |  |
| Arithmetic | Optional |  | Optional |  |  |  | Optional |  |
| **Perceptual Reasoning Index (PRI)** |  |  |  |  |  |  |  |  |
| Picture Completion |  |  | Optional |  |  |  | Optional |  |
| Object Assembly |  |  |  |  | Optional |  | Required |  |
| **Performance Intellectual Quotient (PIQ)** |  |  |  |  |  |  | Required |  |
| Working Memory Index | Required |  | Required |  | Required |  |  |  |
| Digit Span | Required | **✓** | Required | **✓** |  |  |  |  |
| Picture Span | Required |  |  |  |  |  |  |  |
| Picture Memory |  |  |  |  | Required | **✓** |  |  |
| Letter-Number Sequencing | Optional |  | Required | **✓** |  |  |  |  |
| Zoo Locations |  |  |  |  | Optional |  |  |  |
| **Processing Speed Index (PSI)** |  |  |  |  |  |  |  |  |
| Coding | Required | **✓** | Required | **✓** |  |  | Required | **✓** |
| Symbol Search | Required |  | Required | **✓** |  |  | Required |  |
| Cancellation | Optional |  | Optional |  | Optional |  | Optional |  |
| Bug Search |  |  |  |  | Required | **✓** |  |  |

Note: Subtests required for calculation of the FSIQ are indicated by **✓**.

Abbreviations: FSIQ: Full Scale Intelligence Quotient; WPPSI: Wechsler Preschool and Primary Scale of Intelligence; WISC: Wechsler Intelligence Scale for Children.

**SI.2. Oral and written language batteries complementary information**

Oral and written language competencies of the study participants were evaluated through three distinct assessment batteries: EVALEO [Assessment of written and oral language], EXALANG [Language assessment] and BALE [Analytical review of written language]. Table SI.2.1 details the number of children assessed with each battery.

Table SI.2.1: Distribution of oral and written langage batteries completed by children in the GC, TE, and SLD Groups

| **Group (N)** | **EVALEO** | **EXALANG** | **BALE** |
| --- | --- | --- | --- |
| GC (71) | 71 (100.0%) | 0 (0.0%) | 0 (0.0%) |
| TE (114) | 67 (58.8%) | 28 (24.6%) | 19 (16.7%) |
| SLD (116) | 93 (80.2%) | 13 (11.2%) | 10 (8.6%) |

Abbreviations: GC: Gifted children; TE: Twice-Exceptional; SLD: Specific Learning Disorder; EVALEO: EVAluation du Langage Ecrit et du langage Oral [Assessment of written and oral language]; EXALANG: EXAmen de LANGage [Language assessment]; BALE: Bilan Analytique du Langage Ecrit [Analytical review of written language].

The scores obtained on the speech‑language tests are not expressed using the same metrics across the different language batteries. Scores from the EXALANG and BALE batteries are expressed as standardized z‑scores (mean = 0, SD = 1). Scores from the EVALEO [Assessment of written and oral language] battery are expressed as ordered classes (C) ranging from 1 to 7, each corresponding to contiguous percentile intervals (score < 7th percentile = C1; score between the 7th and 20th percentiles = C2; score between the 21st and 38th percentiles = C3; score between the 39th and 62nd percentiles = C4; score between the 63rd and 80th percentiles = C5; score between the 81st and 93rd percentiles = C6; score > 93rd percentile = C7).

Given that Z-scores from the EXALANG and BALE batteries can be converted into EVALEO percentile interval classes, whereas the reverse (converting percentile interval classes back into Z-scores) is not possible, all performance scores have been transformed into EVALEO percentile interval ranks ranging from 1 to 7, as indicated in the conversion Table SI.2.2.

Table SI.2.2: Conversion of EXALANG and BALE Z-scores into the seven EVALEO percentile rank scores

| **EVALEO classes (C)** | **C1** | **C2** | **C3** | **C4** | **C5** | **C6** | **C7** |
| --- | --- | --- | --- | --- | --- | --- | --- |
| EVALEO classes percentile intervals | < 7 | 7 / 20 | 21 / 38 | 39 / 62 | 63 / 80 | 81 / 93 | > 93 |
| EXALANG and BALE Z-score correspondences | < -1.48 | -1.47 / -0.84 | -0.85 / -0.30 | -0.31 / +0.30 | +0.31 / +0.84 | +0.85 / +1.47 | > +1.48 |
| Children’s percentage per C in the general population | 7% | 13% | 18% | 24% | 18% | 13% | 7% |

Note: As an example, a Z-score of -1.2 achieved on an EXALANG subtest would be transformed into a score of 2, which corresponds to the EVALEO C2 rank score, and then would be processed by the CART algorithm.

Abbreviations: EVALEO: EVAluation du Langage Ecrit et du langage Oral; EXALANG: EXAmen de LANGage]; BALE: Bilan Analytique du Langage Ecrit.

**SI.3. Methodology of Classification and Regression Trees Analysis (CART)**

Classification and Regression Tree (CART) analyses were used to identify the most discriminative threshold on the language tests between the TE group and the control group of GC without SLD, maximizing the balance between sensitivity and specificity. As a non‑parametric approach, CART (Breiman et al., 1984) was particularly well‑suited to the study’s dataset, given the high probability of floor effects in the TE group, and the expecting ceiling effects in the GC group, consistent with previously published findings (Lesecq et al., 2024). These patterns in the language test scores made non‑Gaussian distributions highly probable. Secondly, CART provides a solution in the form of a clear and intuitive decision criterion, making it easy for physicians and psychologists to interpret and apply in clinical practice.

The CART algorithm (Hothorn & Zeileis, 2015) applies recursive binary partitioning to split the population into increasingly homogeneous subgroups. Algorithm evaluates every possible cut-point across all test scores to select the split that maximizes the reduction in Gini impurity. This index (ranging from 0 to 1) measures the "purity" of a node, with 0 indicating that all individuals in a branch belong to the same category. Starting from the full sample (root node), the recursive process is applied to each subsequent child node until further splits no longer enhance the model's performance.

In the study, the CART algorithm was applied to model the binomial dependent variable (TE group vs. GC control group) using ordinal independent variables consisting of language test scores ranging from 1 to 7. Analyses were performed in R using the "partykit" package.

Breiman, L., Friedman, J. H., Olshen, R. A., & Stone, C. J. (1984). Chapman & Hall/CRC. *Classification and Regression Trees.* Monterey, CA: Wadsworth, Brooks & Cole. doi:10.1201/9781315139470

Hothorn T., & Zeileis A. (2015). Partykit: A Modular Toolkit for Recursive Partytioning in R. *J Mach Learn Res*. 16(118):3905-30909. doi:10.5555/2789272.2912120

R Core Team. R: A Language and Environment for Statistical Computing. R Foundation for Statistical Computing. https://www.R- project.org/. 2013.

**SI.4. Relative Risks for language tests in SLD and TE groups for the usual threshold (≤ 7^th^ percentile)**

*Notes:* RR for the TE group (RR_TE_) = (proportion of TE children scoring below the 7th percentile) / (proportion of children scoring below the 7th percentile in the general population).

*Notes:* RR for the SLD group (RR_SLD_) = (proportion of SLD children scoring below the 7th percentile) / (proportion of children scoring below the 7th percentile in the general population).

*Notes:* For example, the point for word repetition, with an RR_TE_ on the ordinate of 3.2 and an RR_SLD_ on the abscissa of 8.3, indicated that there were 3.2 times more children scoring below the 7th percentile in the TE group than in the general population, and 8.3 times more children scoring below the 7th percentile in the SLD group than in the general population. Points above the diagonal line indicate that the TE group has a higher proportion of children scoring below the 7th percentile than the SLD group, whereas points below the diagonal line indicate that the TE group has a lower proportion of children scoring below the 7th percentile than the SLD group.

Abbreviations: RR: Relative Risk; SLD: Specific Learning Disorder; TE: Twice-Exceptional.

The RR intersection for the TE and SLD groups was close to the diagonal for speed in Rapid Automatized Naming, indicating that the proportion of children scoring below the standard threshold (7th percentile) was quite similar in both groups (RR_SLD_ = 6.2, RR_TE_ = 5.7). All other language test points were positioned far below the diagonal, indicating that, for these tests, the proportion of children scoring below the 7th percentile was higher in the SLD group than in the TE group relative to the general population. RRs were significantly higher (p < .001) in the SLD group (mean RR_SLD_ = 11.2, range 8.3–13.3) than in the TE group (mean RR_TE_ = 6.7, range 2.9–10.4).

RRs were strongly correlated between TE and SLD groups (R = +0.94, p < .001) (the rapid automatic naming outlier was excluded from the regression). The linear regression of RRs indicated a graded pattern of impairment across language domains in both the TE and SLD groups: mild to moderate impairment on tests assessing prerequisite reading skills, moderately severe on reading tests, and marked to severe impairment on spelling tests.

The gradient of deficit across language domains was more pronounced in the TE group than in the SLD group (linear regression slope = +1.51) indicating that the deficit gap between the SLD and TE groups diminished as one moved along the gradient (ratio RR_SLD_/RR_TE_ = 2.63, for prerequisite reading skills, 1.54 for reading tests and 1.28 for spelling tests).
